# Cochlear Implant Receiver Location and Migration: Experimental Validation Pilot Study of a Clinically Applicable Screening Method

**DOI:** 10.3389/fsurg.2019.00078

**Published:** 2020-01-15

**Authors:** Laura M. Markodimitraki, Inge Stegeman, Adriana L. Smit, Hans G. X. M. Thomeer

**Affiliations:** ^1^Department of Otorhinolaryngology-Head and Neck Surgery, University Medical Centre Utrecht, Utrecht, Netherlands; ^2^University Medical Center Utrecht Brain Center, University Medical Center Utrecht, Utrecht, Netherlands

**Keywords:** cochlear implants, cochlea, validation studies, cochlear implantation, pilot projects, sensorineural hearing loss, neurotology

## Abstract

**Objectives:** Postoperative follow-up after cochlear implantation lacks a reliable screening method to detect cochlear implant receiver device migration. This study aims to validate a clinically applicable method to assess the position and migration of the cochlear implant receiver device.

**Study design:** Validation study.

**Setting:** Tertiary university medical center.

**Participants and method:** To assess the cochlear implant receiver device location, round markers representing the external magnet were placed on both sides of the head of volunteers. Four independent clinicians took measurements of the distances between reference points on the head and the center of the marker. The reference points were: the lateral canthus (LC), tragus tip (TT), the mastoid angle (MA), and the mandibular angle (AM).

**Main outcome measures:** The inter-clinician reliability was determined by calculating the intraclass correlation coefficient (ICC) and confidence interval (CI) with a two-way mixed model and both consistency and absolute agreement types for each distance.

**Results:** Eight volunteers were included resulting in 16 individual cases. The consistency type ICC's for each reference point were: LC 0.90 (CI = 0.80, 0.96), TT 0.83 (CI = 0.69, 0.93), MA 0.75 (CI = 0.56, 0.89), and AM 0.29 (CI = 0.05, 0.59). The absolute agreement ICC's were: LC 0.87 (CI = 0.73, 0.95), TT 0.83 (CI = 0.68, 0.93), MA 0.68 (CI = 0.42, 0.86), and AM 0.18 (CI = 0.01, 0.46). The inter-clinician reliability was good to excellent for the lateral canthus and tragus tip reference points.

**Conclusions:** The cochlear receiver device location can be assessed reliably by measuring the distance between the LC, TT, and the external magnet. This method can be used to registrate implant receiver location after implantation and detect implant migration postoperatively.

## Introduction

Cochlear implantation, first attempted in the 1970's, provides hearing through electrical stimulation for patients with sensorineural hearing loss (1). Nowadays it is a reliable and safe procedure (2–4). The advances in technology and the refinement of surgical techniques (even with broadening indication and age range) have led to low complication rates (1, 5). Amongst reported major complications are device failure, infection/wound complications, electrode and device migration, some of which require revision surgery (5, 6).

Electrode migration has been a subject of recent interest resulting in studies exploring the possibilities of imaging for accurate measurement of the electrode position (7–9). Yet, receiver/stimulator (R/S) device migration is a less explored topic. Recent studies concerning either cochlear implantation complications or fixation techniques, report a R/S migration incidence of 0.0–0.7% (1, 2, 10–12). This migration can result in various complaints: pain (e.g., by contact between the behind-the-ear device and the implant), tension headache, interaction with wearing eyeglasses, which can lead to device failure (2, 3, 10). Failed fixation can result in R/S device migration and it has been suggested that inappropriate device positioning could negatively impact migration (2, 13, 14). Conventionally, the device is positioned roughly in the region supero-posteriorly from the pinna in an angle around 45–60° from the Frankfurt line (15). In the past years several techniques have been described to fixate the implant. These include drilling a bony bed (with or without a canal directing the electrode array toward the mastoid cavity) and tight sutures to stabilize the implant or a screw fixation system (16). Recent scientific reports showed that solely a tight subperiostal pocket might be sufficient to position the implant without any further drilling of the bone of the temporal cortex (10, 17). Attempts have been made to establish the safety of certain fixation techniques by reporting complications. However, none of these studies use objective and validated tools to assess the position and possible migration of the R/S device. Additionally, long term follow-up is often missing.

The possible negative consequences and the lack of objective assessment in the literature of device migration underline the need for a validated and robust method to detect R/S device migration. Only a few studies have valuated methods to objectively assess the exact location of the R/S direct postoperatively and during follow up. Two studies have recently introduced a method to evaluate migration of the R/S using different reference points (18, 19). Other studies have used imaging like computed tomography (CT) to determine the position of the R/S device (13, 20). These studies lack methodological and statistical strength to prove reliability of the proposed measurement method (18, 21). Additionally, the proposed techniques are time-consuming, expensive, and provide radiation-exposure that might be seen as too much a burden for cochlear implanted patients without complaints. We opted for a more patient-friendly, inexpensive and practical method. To determine the exact location of the R/S device on the scalp we developed a model to measure the distance between anatomical reference points and the cochlear implant transmitter. With this study we aim to validate this method to assess the position of the cochlear implant R/S device.

## Materials and Methods

### Ethical Considerations

All procedures performed in this study involving human participants were in accordance with the ethical standards of the institutional and/or national research committee and with the 1964 Helsinki declaration and its later amendments or comparable ethical standards. Written informed consent was obtained from all participants. The Medical Research Ethics Committee of the University Medical Center Utrecht (WAG/mb/19/025018) officially declared this study exempt from official approval as the Medical Research Involving Human Subjects Act (WMO) does not apply.

### Study Design

**Figure 1:**
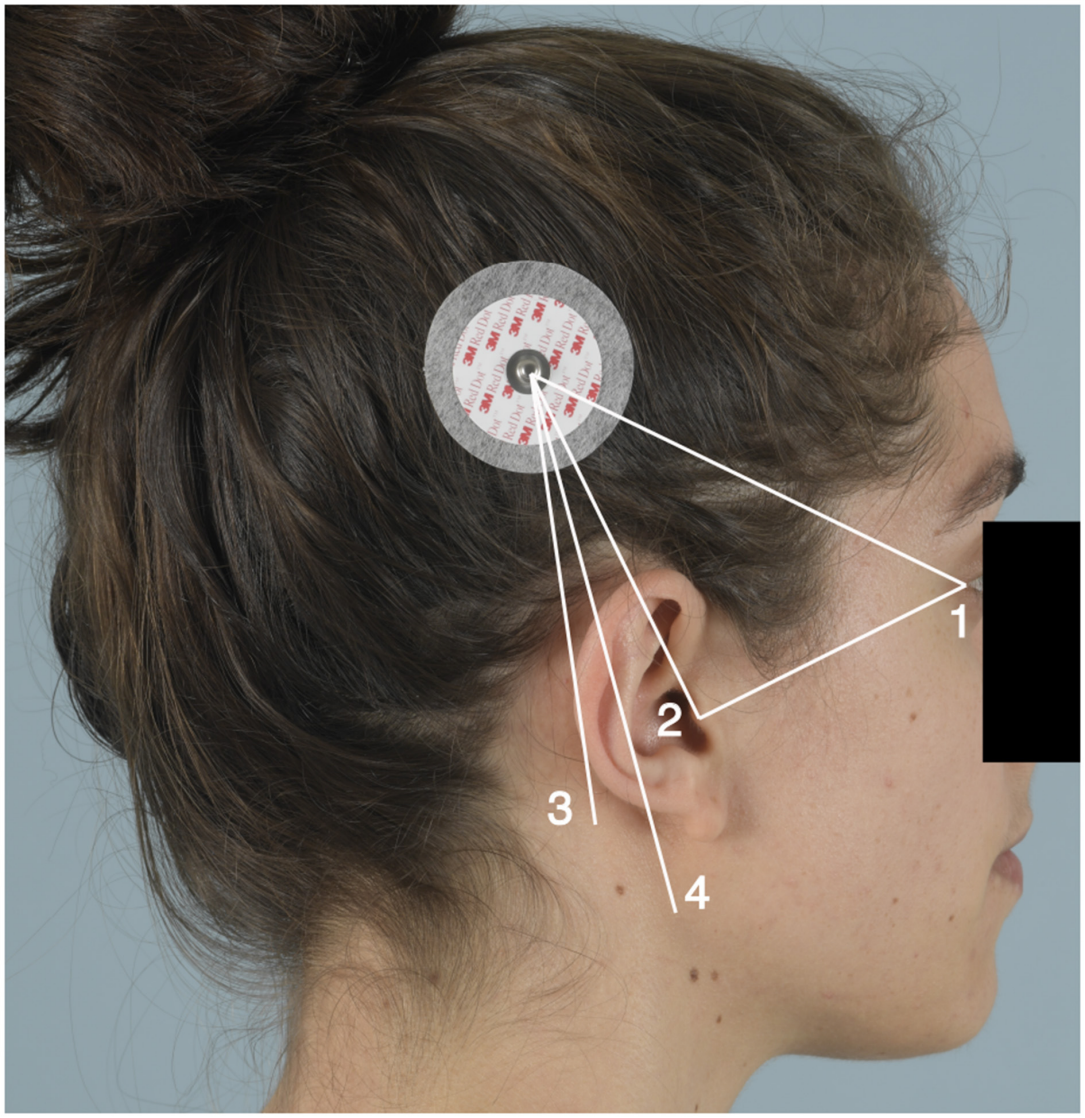
An adhesive marker was placed postero-superiorly of the ear. The numbers represent the reference points: 1, lateral canthus (LC); 2, the tip of the tragus (TT); 3, the mastoid angle (MA); and 4, the mandibular angle (AM). Written consent was obtained from the individual for the use of this image.

A pilot study was conducted to design and validate a measuring method to assess the position of the cochlear implant R/S device. For this proof-of-concept study we used healthy volunteers. As the transmitter connects to the R/S device by the internal magnet, the transmitter's center was used as a reference point to determine the position of the R/S device externally. We measured the distance between certain anatomical reference points and the transmitter. The transmitter magnet was represented by a round adhesive marker placed postero-superiorly of the ear. These markers were placed on both sides of the head of the volunteer directly on the scalp (if the volunteer was bald) or on the hair (which was pulled into a bun) (see Figure 1). Placement of the markers was done at random. Four clinicians with variable expertise in the field of cochlear implantation surgery, namely two members of staff, one fellow, and one medical student, took the assigned measurements independently. They were given written instructions and carried out the measurements without any additional training. The use of volunteers allowed a high number of raters and sequential measurements under similar conditions using both sides of the head. Either side of each volunteer's head were seen as two individual cases.

### Measurement Method

The reference points chosen are the lateral canthus (LC), the tip of the tragus (TT), the mastoid angle (MA), and the mandibular angle (AM). Measurements between those points and the center of the adhesive marker were taken, as well as the distance between the lateral canthus and the tragus tip as a control measurement. A flexible measuring tape was used.

### Statistical Analysis

Data were analyzed using IBM SPSS Statistics for Windows (version 21.0; IBM Corp., Armonk, NY, USA). Measurements between the LC, TT, MA, AM, and the marker as well as between the LC and TT were evaluated. We used different reference points due to expected variation of measurement accuracy. To determine reliability between reference points and suitability for clinical practice, intraclass correlation coefficients (ICC) (single measures) with 95% confidence interval (CI) were calculated. This was executed via a two-way mixed model and both consistency and absolute agreement type. We considered ICC values <0.4 indicative of poor reliability. Values between 0.4 and 0.75 indicative of moderate reliability, 0.75 and 0.90 indicative of good reliability and ICC values ≥ 0.90 indicative of excellent reliability. These thresholds are based on existing literature. However, the ICC should be interpreted with the sample variability in mind. Therefore, we calculate the range of measurement per distance to illustrate the homogeneity of the subjects. Small inter-subject variability results in a depress of the ICC (22). Means and standard deviation were calculated for each case per distance and for each clinician per distance. This study will be reported according to the guidelines for reporting reliability and agreement and according to the STROBE statement (23).

## Results

A total of nine volunteers were measured by four clinicians on both sides of the head. One volunteer was excluded from the study. The adhesive marker of the excluded volunteer was displaced during the measurements due to the hair bun coming loose before measurements could be completed. This resulted in a total of 16 individual cases (see Appendix). The range of the measurements per distance were as follows: LC to marker 130–169 mm, TT to marker 75–100 mm, MA to marker 65–111 mm, AM to marker 113–158 mm, and LC to TT 73–92 mm (Table 1). The standard deviation of the mean range calculated for each case per distance was as follows: LC to marker 0.5–5.2 mm, TT to marker 1.0–6.2 mm, MA to marker 1.6–10.2 mm, AM to marker 2.4–13.7 mm, and LC to TT was 1.3–6.2 mm. Furthermore, the standard deviation extracted from all clinicians per distance was between 3.1 and 9.5 mm (Table 1).

**Table 1:** Measurements per distance (mm) for all raters and ratios of measurement.

**Raters**	**Median (range)**				
	**LC to magnet**	**TT to magnet**	**MA to magnet**	**AM to magnet**	**LC to TT**
A	147.5 (132–169)	88.5 (76–100)	94.0 (75–110)	134.0 (120–141)	85.0 (80–92)
B	148.0 (132–160)	88.5 (75–100)	93.0 (83–101)	140.0 (130–154)	84.0 (75–89)
C	147.5 (133–163)	89.0 (73–100)	92.5 (80–111)	137.5 (120–158)	85.0 (80–92)
D	145.5 (128–159)	88.0 (70–98)	89.5 (65–108)	126.7 (113–146)	79.5 (73–87)

*LC, lateral canthus; TT, tragus tip; MA, mastoid angle; AM, mandibular angle; SD, standard deviation*.

The ICC's regarding the various distances calculated with a consistency type and absolute agreement type are found in Table 2. The ICC's of LC to marker and TT to marker for both absolute agreement and consistency type are good to excellent. Whereas, MA to marker and AM to marker ICC's are moderate to poor.

**Table 2:** Range of measurements per distance (mm) and intra-class correlation coefficient with 95% confidence interval.

**Measured distance**	**Median (range)**	**Consistency type**		**Absolute agreement type**	
		**ICC**	**95% CI**	**ICC**	**95% CI**
LC to marker	148.0 (130–169)	0.90	[0.80, 0.96]	0.87	[0.76, 0.93]
TT to marker	89.0 (75–100)	0.83	[0.69, 0.93]	0.74	[0.55, 0.88]
MA marker	92.0 (65–111)	0.75	[0.56, 0.89]	0.65	[0.44, 0.83]
AM to marker	135.0 (113–158)	0.29	[0.05, 0.59]	0.26	[0.04, 0.55]
LC to TT	84.5 (73–92)	0.50	[0.25, 0.74]	0.47	[0.24, 0.71]

*LC, lateral canthus; TT, tragus tip; MA, mastoid angle; AM, mandibular angle; SD, standard deviation; ICC, intra-class correlation coefficient; CI, confidence interval*.

## Discussion

As device migration can result in major difficulties for the patient, which in some cases necessitates revision surgery, detection of migration can be of value for the patient. However, simple and validated techniques are missing. With this study, we aimed to validate a method to easily assess the position of R/S device by measuring distances between the magnet of the transmitter and certain anatomical reference points. The ICC's found for the distances LC to marker and TT to marker indicate good to excellent reliability, also considering the 95% confidence interval. By this, the here presented screening method using these distances can be used to determine device position postoperatively. Clinicians should compare the results of the measurements from each outpatient clinic visit in order to detect gradual changes as a possible indication of migration.

In our study the mean of the measurement differences between raters for the distances LC to magnet and TT to magnet were 5.3 mm (SD ± 2.8) and 5.9 mm (SD ± 2.5), respectively (see Appendix). In a previous study by Maxwell et al. about R/S migration, measurement differences exceeding 5 mm were proposed as true migration. they reported a migration of the R/S device in 25.9% of the implants within the first 6 months postoperatively (*p* = 0.43) (24). Though, by the lack of using a validated measurement method in the study and without any consensus regarding a clinically relevant R/S device migration no conclusions can be made which cut off values must be met to be defined as “true migration.”

In recent years, assessment of the precise position of the R/S of the cochlear implant seems to gain momentum as a topic of international interest (13, 18, 20). There is need for a validated measurement method that can be integrated in routine outpatient clinical follow up. This method provides clinicians with an objective and easy to use tool to detect migration. In addition to clinical use, this method could provide an objective tool to report reliability of device fixation techniques and quality of care in the literature. We suspect device migration to be an underreported clinical parameter after cochlear implantation. This can be demonstrated by the study of Lui et al. They reported slight device migration in all included patients when objectively assessing the R/S position and potential migration by using CT scanning (mean ± SD; 2.1 ± 1.4 mm) (13). Although, these migrations would not have been detected by our measurement method, it is noteworthy that this objective method detected R/S migration in all included patients.

In this study, we did not provide the clinicians with any training before measuring the volunteers. This could lead to differences in measuring technique between clinicians (as seen in Table 1), but it did not lower the inter-clinician reliability of the method.

### Strengths and Limitations

To the best of our knowledge, this is the first validation study of a measurement method for the assessment of the R/S device position that is low-cost, easy to apply, does not expose the patient to radioactive environment and can be used during follow-up in an outpatient setting. Until now there is no validated measurement tool with which comparison of measurements is possible. Additionally, patients sometimes are seen by different clinicians in which inter-rater reliability can influence outcome which is taken in account in the presented study. The measurement method in this study is validated using recommended statistical analysis (21, 22). One limitation of the technique is the need to pull the flexible measurement tape over the pinna, which could influence the accuracy of the measurements. However, the ICC from this measurement from the TT to the marker was satisfactory. A limitation of this study is that we chose to do this pilot study on a small group of healthy individuals, using markers rather than cochlear transmitters to carry out the measurements. The adhesive markers had a clear center unlike the transmitter magnet. The marker was fixated on the hair rather than the scalp, resulting in unwanted marker mobility. This can be demonstrated by the excluded volunteer. During the measurements of this volunteer it was clear that misplacement of the adhesive marker had taken place due to loosening of the hair. Positional shifts due to hair movement could have occurred also to other volunteers but none was detected at the time of the measurements. However, in a clinical setting the transmitter is attached firmly on the scalp thus eliminating this factor. Finally, this method overcomes the problem of the different processor styles.

### Future Prospective

The provided measurement method infers future investigation in implanted patients to extrapolate the results in real-life cochlear implant users and relate outcome to fixation techniques. Establishment of a reference standard regarding implant migration assessment is necessary as well as proper postoperative follow-up to detect the device location and potential migration. The relation between implant position and migration and subjective patient experience of this outcome should be part of this investigation.

## Conclusion

Measuring distances between the lateral canthus, the tragus tip and the marker as a proxy for the transmitter magnet of a CI, as described in this study, is a reliable method to assess the position of the R/S device. The technique could be implemented during follow-up of cochlear implant patients as an easy to use, radiation-free tool to screen for migration. The next step would be the validation of this method in cochlear implant patients and the relation between migration and subjective quality of life outcome assessment.

## Data Availability Statement

The datasets generated for this study are available on request to the corresponding author.

## Ethics Statement

The participants provided their written informed consent to participate in this study. Written informed consent was obtained from the individual(s) for the publication of any potentially identifiable images or data included in this article.

## Author Contributions

LM and AS performed the experiments. LM performed the calculations and wrote the manuscript with input from all authors. LM, IS, AS, and HT were involved in the conception and design of the study, provided critical feedback, and helped shape the analysis and final manuscript.

### Conflict of Interest

The authors declare that the research was conducted in the absence of any commercial or financial relationships that could be construed as a potential conflict of interest.
