# Supplementary material for: Cochlear Implant Receiver Location and Migration: Experimental Validation Pilot Study of a Clinically Applicable Screening Method
Source: Front Surg. 2020 Jan 15;6:78. doi: 10.3389/fsurg.2019.00078 (PMC6974530; doi:10.3389/fsurg.2019.00078)
Supplement: Supplementary file 1 [file Table_1.DOCX]

Supplementary Material

Appendix Measurements per case for all raters and ratios of measurement. a= right side of the head, b= left side of the head, LC= lateral canthus, TT= tragus tip, MA= mastoid angle, AM= mandibular angle, SD= standard deviation.

| Cases | LC to magnet | | TT to magnet | | MA to magnet | | AM to magnet | | LC to TT | |
| --- | --- | --- | --- | --- | --- | --- | --- | --- | --- | --- |
|  | Mean ± SD | Median (range) | Mean ± SD | Median (range) | Mean ± SD | Median (range) | Mean ± SD | Median (range) | Mean ± SD | Median (range) |
| 1a | 147.3 ± 2.2 | 147.0 (145-150) | 92.3 ± 2.2 | 92.0 (90-95) | 95.0 ± 1.6 | 95.0 (93-97) | 139.8 ± 9.5 | 135.0 (135-154) | 83.0 ± 2.0 | 84.0 (80-84) |
| 1b | 151.5 ± 1.7 | 151.5 (150-153) | 93.5 ± 1.0 | 94.0 (92-94) | 89.8 ± 3.7 | 90.0 (85-94) | 131.8 ± 7.1 | 131.5 (124-140) | 81.5 ± 2.7 | 81.0 (79-85) |
| 2a | 138.3 ± 2.4 | 139.0 (135-140) | 76.8 ± 3.5 | 75.0 (75-82) | 85.5 ± 4.2 | 85.5 (81-90) | 127.8 ± 8.9 | 129.5 (117-135) | 79.5 ± 5.8 | 79.5 (74-85) |
| 2b | 132.3 ± 0.5 | 132.0 (132-133) | 78.0 ± 3.2 | 77.5 (75-82) | 85.3 ± 4.3 | 84.0 (79-88) | 128.5 ± 9.2 | 129.0 (118-138) | 78.5 ± 3.7 | 80.0 (73-81) |
| 3a | 152.5 ± 3.3 | 153.5 (148-155) | 90.3 ± 3.2 | 89.0 (88-95) | 99.8 ± 5.0 | 100.5 (93-105) | 137.0 ± 5.4 | 136.5 (132-143) | 85.8 ± 3.0 | 85.0 (83-90) |
| 3b | 151.0 ± 2.9 | 151.5 (147-154) | 84.3 ± 1.7 | 84.5 (82-86) | 86.3 ± 3.5 | 86.5 (82-90) | 129.0 ± 7.6 | 126.5 (123-140) | 86.8 ± 4.6 | 87.0 (81-92) |
| 4a | 147.3 ± 3.0 | 148.0 (143-150) | 90.8 ± 2.2 | 91.0 (88-93) | 91.0 ± 3.4 | 91.0 (88-96) | 137.0 ± 7.3 | 137.5 (128-145) | 85.3 ± 5.2 | 87.0 (78-89) |
| 4b | 137.3 ± 5.2 | 139.0 (130-141) | 84.5 ± 1.9 | 84.0 (83-87) | 85.0 ± 3.2 | 85.5 (81-88) | 129.5 ± 11.9 | 132.0 (113-141) | 84.0 ± 2.9 | 84.5 (80-87) |
| 5a | 143.3 ± 3.7 | 144.5 (138-146) | 89.8 ± 6.2 | 88.5 (84-98) | 95.5 ± 7.1 | 96.5 (86-103) | 133.3 ± 8.0 | 130.5 (127-145) | 84.0 ± 4.7 | 83.5 (79-90) |
| 5b | 147.0 ± 2.3 | 147.0 (145-149) | 85.75 ± 1.9 | 86.5 (83-87) | 86.5 ± 3.7 | 86.0 (83-91) | 128.0 ± 8.5 | 130.5 (116-135) | 83.5 ± 1.3 | 83.5 (82-85) |
| 6a | 141.8 ± 2.8 | 141.5 (139-145) | 96.5 ± 1.3 | 96.5 (95-98) | 106.5 ± 3.1 | 106.5 (103-110) | 145.5 ± 9.0 | 145.0 (135-157) | 86.8 ± 5.2 | 89.0 (79-90) |
| 6b | 147.5 ± 1.3 | 147.5 (146-149) | 87.8 ± 1.5 | 88.0 (86-89) | 93.8 ± 2.9 | 92.5 (92-98) | 132.3 ± 9.4 | 137.0 (124-143) | 89.8 ± 2.6 | 90.0 (87-92) |
| 7a | 162.0 ± 4.7 | 160.0 (159-169) | 97.0 ± 2.2 | 96.5 (95-100) | 94.3 ± 3.0 | 94.0 (91-98) | 133.8 ± 3.0 | 134.0 (130-137) | 82.5 ± 4.9 | 82.5 (77-88) |
| 7b | 145.0 ± 2.5 | 144.5 (143-148) | 88.8 ± 2.5 | 88.5 (86-92) | 98.0 ± 3.7 | 98.5 (93-102) | 138.8 ± 7.1 | 140.0 (129-146) | 83.5 ± 1.7 | 83.0 (82-86) |
| 8a | 159.5 ± 2.7 | 159.0 (157-163) | 98.8 ± 1.9 | 99.5 (96-100) | 101.5 ± 8.4 | 101.0 (93-111) | 142.3 ± 12.4 | 140.5 (130-158) | 85.3 ± 2.1 | 85.0 (83-88) |
| 8b | 153.3 ± 3.1 | 154.0 (149-156) | 87.3 ± 3.2 | 87.5 (84-90) | 78.0 ± 10.2 | 79.5 (65-88) | 130.8 ± 13.7 | 130.5 (118-144) | 84.0 ± 6.2 | 86.5 (75-88) |
